# MicroRNAs as predictors for CNS relapse of systemic diffuse large B-cell lymphoma

**DOI:** 10.18632/oncotarget.20902

**Published:** 2017-09-15

**Authors:** Nir Pillar, Osnat Bairey, Neta Goldschmidt, Yakov Fellig, Yevgenia Rosenblat, Itchak Shehtman, Danielle Haguel, Pia Raanani, Noam Shomron, Tali Siegal

**Affiliations:** ^1^ Sackler Faculty of Medicine, Tel Aviv University, Tel Aviv, Israel; ^2^ Institute of Hemathology, Davidoff Institute of Oncology, Rabin Medical Center, Petach Tikva, Israel; ^3^ Department of Hemathology, Hadassah Hebrew University Medical Center, Jerusalem, Israel; ^4^ Department of Pathology, Hadassah Hebrew University Medical Center, Jerusalem, Israel; ^5^ Department of Pathology, Rabin Medical Center, Petach Tikva, Israel; ^6^ Department of Pathology, Meir Medical Center, Kefar Saba, Israel; ^7^ Neuro-Oncology Center, Davidoff Institute of Oncology, Rabin Medical Center, Petach Tikva, Israel

**Keywords:** systemic DLBCL, CNS relapse prediction, microRNA, miR-20a, miR-30d

## Abstract

Systemic diffuse large B-cell lymphoma (DLBCL) is a potentially curable disease using current regimen of immunochemotherapy. Central nervous system (CNS) relapse is a complication that occurs in approximately 5% of DLBCL patients and is associated with a high fatality rate. Early identification of molecular markers for CNS involvement may serve for the highly needed accurate stratification of patients into risk groups regarding CNS relapse. MicroRNAs (miRNAs) are small non-coding RNA molecules that regulate gene expression at the post-transcriptional level and are known to be involved in DLBCL pathophysiology. In this study, we utilized miRNA multiplex reading of systemic newly diagnosed DLBCL samples obtained from patients with clinical risk factors for CNS involvement whose disease course was distinguished by the presence or absence of subsequent CNS relapse. The analysis detected two differentially expressed miRNAs, miR-20a and miR-30d, that predict for CNS involvement. Replication of these results in different samples was used for validation. We performed bioinformatics miRNA-target enrichment analysis to reveal a number of putative mechanisms for these miRNAs regulation of CNS relapse, including neuronal plasticity and WNT signaling pathway. Altogether, we show that the expression level of two miRNAs may have valuable information that may refine stratification for patients-at-risk for relapse with CNS involvement in DLBCL. Further larger scale studies are needed to shed light on the pathways involved in this disease.

## INTRODUCTION

Diffuse large B-cell lymphoma (DLBCL) is the most common non-Hodgkin lymphoma (NHL). It represents a heterogeneous group of tumors with a high variance of genetic abnormalities, clinical features, response to treatment and prognosis [1]. Treatment outcome of DLBCL is associated with clinical factors that comprise the prognostic tools known as the international prognostic index (IPI) and its variants. However, all available prognostic tools fail to reliably predict the clinical course in many patients and to accurately anticipate central nervous system (CNS) involvement.

CNS relapse is a devastating and often fatal complication that occurs in up to 7% of patients with DLBCL [2, 3]. The grim prognosis associated with CNS relapse and the observation that it usually occurs within 2 years from initial diagnosis argue for a prophylactic approach [4]. Commonly used strategies for CNS prophylaxis include interventions that carry additional risk of adverse events, including systemic and neurologic toxicity. Currently prophylactic treatment is given to patients considered to be at high risk for CNS relapse based on clinical risk stratification. The baseline risk factors associated with increased risk of CNS relapse include high IPI, high LDH serum levels, more than one extranodal sites of disease, site-specific disease such as testes, uterus, breast, paranasal or parameningeal, and bone marrow. involvement [2-6]. However, clinical stratification fails to identify a large proportion of patients that will relapse in the CNS [4]. The lack of a reliable stratification results in significant practice variability with regards to application of prophylactic therapy. As a result, a large number of patients who will never develop CNS relapse are currently exposed to a potentially toxic prophylactic regimen while other patients who will actually develop CNS involvement do not receive prophylactic therapy that might prevent CNS relapse.

The assumption is that the spread of lymphoma cells into the CNS is probably caused by an expansion of unique clones that have propensity to penetrate extranodal sites including the brain and leptomeninges. Early identification of molecular markers of these clones may serve for the highly needed accurate stratification of patients into risk groups regarding CNS relapse.

MicroRNAs (miRNAs) are small non-coding RNA molecules that regulate gene expression at the post-transcriptional level. Expression of individual miRNAs and miRNA signatures is a powerful diagnostic and prognostic tool. Some miRNAs (miR-125a and miR-125b, miR-17-92 cluster and miR-155), were identified as important effectors in the pathogenesis of DLBCL [7]. Other studies proposed models of miRNAs signatures that enabled discrimination between various lymphoid malignancies with high accuracy [8]. Additional investigations identified a series of differentially expressed miRNAs (including 4 miRNAs of the miR-17-92 cluster) as being able to differentiate between subtypes of DLBCLs [9]. Furthermore, it was also found that miRNAs may predict survival and progression free survival of DLBCL (miR-18a, miR-181a and miR-222) independently of clinical factors [10]. So far, none of the published studies attempted to use miRNAs as potential biomarkers for the propensity to develop CNS relapse in newly diagnosed DLBCL. In the current study, our aim was to search for miRNAs signature that can predict CNS relapse of DLBCL. We based our investigation on global miRNA expression obtained from the original systemic tissue that served for diagnosis of DLBCL. Our patients were divided into two cohorts: those who did not develop CNS involvement vs. those who suffered from CNS relapse.

## RESULTS

Tissue samples of thirty-six patients with systemic DLBCL were selected for this study (see Tables 1 and 2). Of these, 16 belonged to clinical high-risk patients without CNS relapse and 20 to patients with a documented CNS relapsed. The median age of patients without CNS involvement was 65.5 years (range: 38-79) and that of patients with CNS relapse was 60.5 years [range: 38-80 (p=0.67)]. Recently, a CNS prognostic model which incorporates the five IPI factors as well as kidney/adrenal involvement (CNS-IPI) has been proposed [11]. This risk model stratifies DLBCL patients into three risk groups: low risk (LR = 0-1 factors); intermediate risk (IR = 2-3 factors) and high risk (HR = 4-6 factors). We retrospectively added the CNS-IPI risk evaluation to the two study groups (Tables 1 and 2). By this prognostic index the CNS relapse group contained 5% (1/20) LR, 65% (13/20) IR and 30% (6/20) HR patients. Similar proportion of IR and HR patients were included in the study group without CNS relapse: 68.7% (11/16) IR and 31.2% (5/16) HR patients. Systemic relapse was more frequent in the group of CNS involvement and occurred in 35% (7/20) of patients while in the group without CNS relapse 6% (1/16) developed systemic relapse. In addition, 10 brain biopsies of DLBCL (5 samples of relapsed CNS DLBCL and 5 of PCNSL) were included in the first part of the analysis in order to visualize distance and relatedness between systemic vs. CNS DLBCL biopsies when testing for global miRNA expression.

**Table 1:** Characteristics of patients with CNS relapse of systemic DLBCL

Patient No.	Gender	Age at Dx. of DLBCL	Site of diagnostic systemic tissue biopsy	Risk factors for CNS involvementat initial diagnosis			CNS-IPIrisk group (score)	Diagnosis of CNS relapse
				Site specific	Extranodal sites	BM/Para-meningeal involvement		
1	M	59	Testis	Testis	1	no/no	LR (1)	Brain biopsy
2	F	41	Uterus/ovary	Uterus	2	no/no	IR (2)	Brain biopsy
3	F	75	Uterus	Uterus	>2	yes/no	HR (5)	MRIcytology
4	F	75	Retroperitoneal mass		1	no/yes	IR (3)	MRI cytology
5	F	77	Lymph node		1	no/no	IR (2)	MRI
6	F	51	Uterus	Uterus	3	no/no	HR (4)	MRI
7	F	46	Femur		2	no/no	HR (4)	Brain biopsy
8	M	50	Spleen		1	no/no	IR (2)	MRI
9	M	61	Testis	Testis	3	yes/no	IR (2)	MRI cytology
10	F	60	Parotis		2	yes/yes	HR (5)	MRI cytology
11	F	68	Colon		2	no/no	IR (3)	MRI
12	M	40	Testis	Testis	3	no/no	IR (3)	MRI cytology
13	M	72	Spleen		1	yes/no	HR (4)	MRI
14	M	70	Retroperitoneal mass		2	yes/no	IR (3)	MRI
15	F	80	Breast	Breast	1	no/no	IR (3)	MRI cytology
16	M	67	Testis	Testis	1	no/no	IR (2)	MRI cytology
17	M	53	Lymph node		2	yes/no	IR (3)	MRI cytology
18	F	54	Lymph node		2	yes/no	HR (4)	MRIcytology
19	M	38	Abdominal mass		1	yes/no	IR (3)	MRI cytology
20	M	79	Testis	Testis	1	no/no	IR (2)	MRI

Dx. = diagnosis; BM=bone marrow involvement; M=male; F=female; CNS-IPI=central nervous system-international prognostic index; Risk groups: LR=low risk, IR=intermediate risk; HR=high risk.

**Table 2:** Characteristics of patients without CNS relapse of systemic DLBCL

Patient No.	Gender	Age at Dx. of DLBCL	Site of diagnostic systemic tissue biopsy	Risk factors for CNS involvement at initial diagnosis			CNS-IPIrisk group (score)
				Site specific	Extranodal sites	BM / Para-meningeal involvement	
21	F	69	Lymph node		2	yes/no	IR (3)
22	M	38	Liver		2	no/no	IR (3)
23	F	52	Stomach		2	yes/no	IR (3)
24	M	79	Colon		>1	no/no	IR (3)
25	M	73	Thoracic mass		2	no/no	IR (3)
26	F	46	Kidney	Kidney	1	no/no	IR (3)
27	M	61	Lymph node		>1	no/no	IR (2)
28	F	77	Omentum		>1	no/no	IR (3)
29	F	74	Lymph node		3	no/no	IR (3)
30	F	79	Lymph node		3	yes/no	HR (4)
31	F	72	Liver		3	yes/no	HR (5)
32	F	48	Lymph node		2	no/yes	IR (2)
33	M	66	Mediastinal mass		2	no/no	HR (4)
34	F	62	Colon		2	no/no	HR (4)
35	F	65	Gastric antrum		3	no/yes	IR (3)
36	M	43	Liver		3	no/no	HR (4)

Dx. = diagnosis; BM=bone marrow involvement; M=male; F=female; CNS-IPI=central nervous system-international prognostic index; Risk groups: LR=low risk, IR=intermediate risk; HR=high risk.

**Figure 1:**
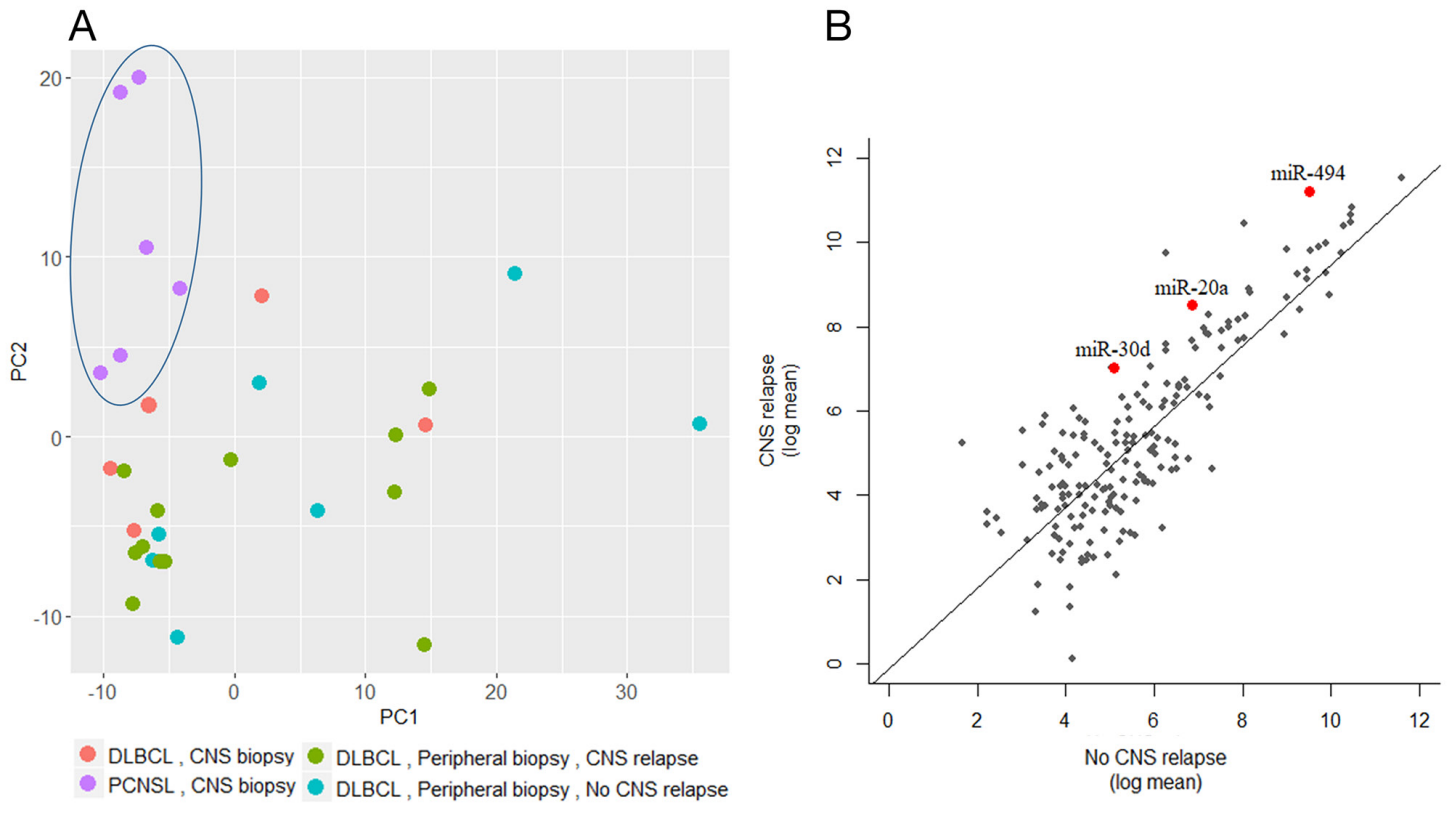
global miRNA expression analysis **(A)** Principal component analysis of 29 samples analyzed via NanoString. Each dot represents the collective expression of all microRNAs in one sample. Each color is indicative of a different lymphoma group. PCNSL samples (purple dots, marked by an ellipse) demonstrate global miRNA expression that differs from the other study groups of systemic lymphomas (green- with subsequent CNS relapse and turquoise- without subsequent CNS relapse) and from brain biopsies of relapsed systemic lymphoma (red dots). **(B)** Log mean expressions of 137 microRNAs that passed quality control filters of NanoString analysis compared between the CNS relapse and the no CNS relapse DLBCL groups. Each dot represents miRNA, red represent miRNA with significant differential expression.

**Figure 2:**
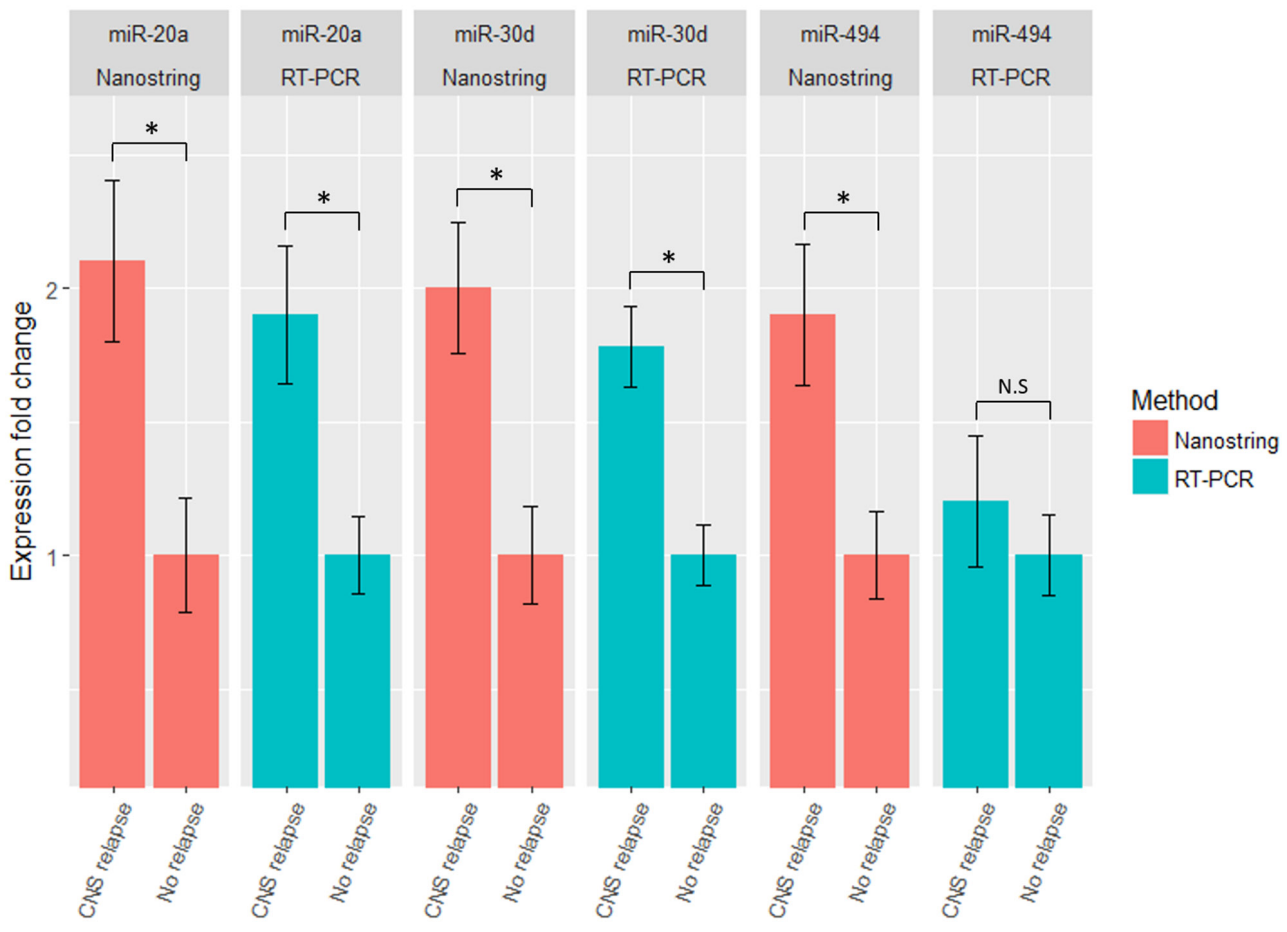
miR-20a, miR-30d and miR-494 relative expression in discovery and replication groups Expression level of miR-20a, miR-30d and miR-494 in testing (NanoString) and validation (RT-PCR) groups. MiR-20a and miR-30d were found to be significantly upregulated in the CNS relapse group by NanoString analysis and these results were validated by testing additional samples using RT-PCR. MiR-494 was not significantly different when testing additional samples. Data are represented as mean +/- SEM.*<0.05

**Figure 3:**
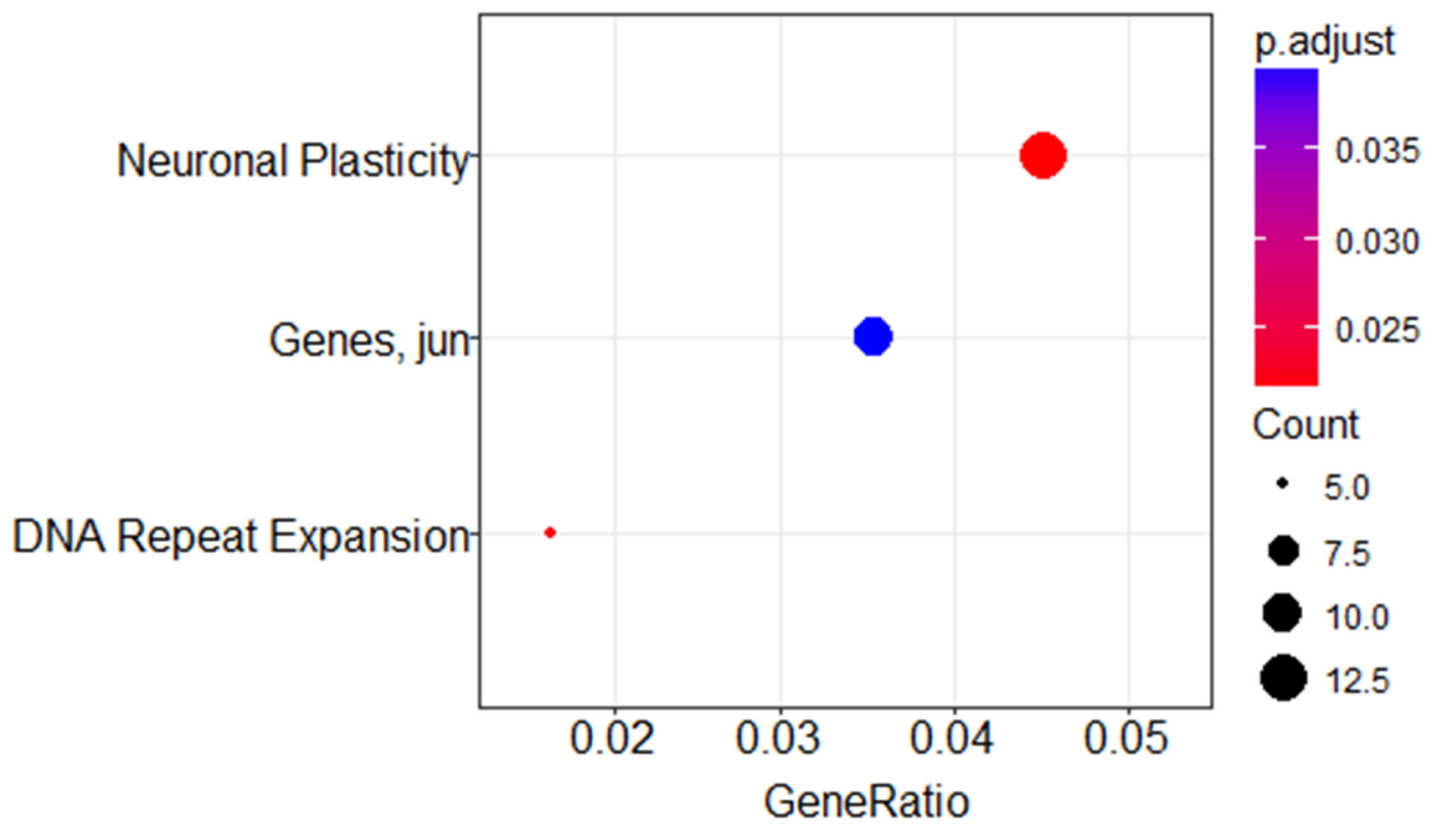
MeSH enrichment analysis using miR-20a and miR-30d predicted targets Dotplot representation of miR-20a and miR-30d predicted targets in Pubmed/Medline databases. GeneRatio is the number of genes participating in the specific process divided by the total number of genes in the miRNA-target list. Dot sizes (“Count”) are relative to the total number of predicted gene targets from the miRNA-target list that are known to participate in the listed processes and dot color indicates statistical significance. Neuronal Plasticity (large red dot) has 13 participating genes included in the miR-target list (*p*=0.02), Jun related pathway (blue dot) has 10 participating genes (*p*=0.039) and DNA Repeat Expansion (small red dot) has 5 participating genes (*p*=0.025).

A minimum amount of 1μg total RNA with high purity values was extracted from all 46 samples. Twenty nine RNA samples (19 systemic biopsies and 10 CNS biopsies) were sent for miRNA profiling via NanoString platform (see Methods for raw data normalization and analysis). We used principal component analysis in order to assess whether a global miRNA expression can distinguish between the four DLBCL groups. Only PCNSL samples were set apart from the other groups while no clear distinction was possible between the 3 systemically originating DLBCL groups (Figure 1A). To evaluate whether a miRNA-based risk classifier pattern can be identified we performed a comparison of miRNA expression between the two systemic DLBCL groups (with CNS relapse vs. no CNS relapse). Several miRNAs were differentially expressed between the two groups (Table 3) and three miRNAs had P<0.05 (miR-20a, miR-30d and miR-494) (Figure 1B). RT-PCR was used to validate the results utilizing additional 17 samples which have not been analyzed by NanoString assay. Samples included 8 cases of DLBCL who developed CNS relapse and 9 cases of DLBCL with no CNS involvement. RT-PCR analyzes for miR-20a, miR-30d and miR-494 verified that MiR-20a and miR-30d demonstrated the same trend as seen in the previous NanoString assay while miR-494 was not differentially expressed (Figure 2). Next, we tested gene enrichment and semantic similarity of miRNA-targets in Kyoto Encyclopedia of Genes and Genomes (KEGG), Gene Ontology (GO) and MeSH (MEDLINE/PubMed indexed articles) database, Using ClusterProfiler R package [12]. For this purpose, we considered genes as miRNA targets if predicted as a target-gene by at least two out of four well-established prediction tools: TargetScan [13], Pita [14], Pictar [15] and Diana [16]. This resulted in 241 and 35 target-genes per miR-20a and miR-30d, respectably (Supplementary Tables 1 and 2). Enrichment and similarity analysis were performed on both miRNA-target genes as a whole, reasoning that the differentially expressed miRNA response act in concert. KEGG and GO analysis revealed several statistically significant enriched pathways related to cancer and axonal development (Table 4 and 5). Significant enrichment for neuronal plasticity and JUN related genes was detected in MeSH database (Figure 3).

**Table 3:** Top differentially expressed miRNAs

microRNA	Mean No Relapse	SE No Relapse	Mean Relapse	SE Relapse	P-value
**hsa-miR-20a**	115.51	38.46	362.24	120.40	**0.02**
**hsa-miR-30d**	32.77	10.76	129.77	39.08	**0.02**
**hsa-miR-494**	833.72	150.77	2379.51	732.62.	**0.02**
hsa-miR-188	25.07	8.59	71.93	29.24	0.07
hsa-miR-574	247.47	47.07	140.32	25.50	0.08
hsa-miR-146a	624.69	165.37	336.91	72.43	0.10
hsa-miR-630	77.30	31.75	855.74	737.38	0.12
hsa-miR-302d	75.37	25.34	201.43	76.90	0.12

**Table 4:** KEGG enriched pathways targeted by miR-20a/miR-30d

Description	p-value	#genes
Pathways in cancer	4.19E-05	22
Protein processing in endoplasmic reticulum	0.001096	16
Lysine degradation	0.00727	4
Ubiquitin mediated proteolysis	0.007741	14
mRNA surveillance pathway	0.042026	9
N-Glycan biosynthesis	0.042353	3
Wnt signaling pathway	0.042353	11
Proteoglycans in cancer	0.042353	13
Glioma	0.042353	6

**Table 5:** GO enriched pathways targeted by miR-20a/miR-30d

ID	Description	p-value	#genes
GO:0007409	axonogenesis	6.12E-06	19
GO:0048667	cell morphogenesis involved in neuron differentiation	9.07E-06	21
GO:0002064	epithelial cell development	1.86E-05	12
GO:0061564	axon development	1.97E-05	19
GO:0051656	establishment of organelle localization	8.41E-05	17
GO:0001885	endothelial cell development	8.56E-05	6

## DISCUSSION

In this study we utilized miRNA multiplex reading of systemic newly diagnosed DLBCL samples obtained from patients with clinical risk factors for CNS involvement whose disease course was distinguished by the presence or absence of subsequent CNS relapse. We detected two differentially expressed miRNAs, miR-20a and miR-30d, that predict for impending CNS involvement. These findings strongly support the assumption that CNS relapse is probably caused by an expansion of unique clones that have propensity to penetrate the brain and leptomeninges and that early identification of molecular markers of these clones may serve for accurate risk stratification regarding prospects for CNS relapse.

The recently reported CNS-IPI clinical risk model [11] retrospectively identified three risk groups for CNS involvement with a predicted 2-year CNS relapse rate of less than 1% for LR, 3-5% for IR and 10-16% for HR patients. This study did not evaluate whether CNS relapse occurred in isolation or with systemic disease. Another report [17] that used the CNS-IPI stratification found similar CNS relapse rates and noted that 56% of the patients had an isolated CNS involvement while 44% developed systemic relapse as well. Once our two study groups are appraised by CNS-IPI index (Table 1 and 2) it confirms that the proportion of IR and HR groups is similar in both cohorts. The 35% systemic relapse rate observed in the group with CNS involvement is similar to the previous report [17]. Yet, the observed difference in miRNAs expression indicates that molecular markers may have an added value for identification of patients-at-risk for relapse and particularly for CNS relapse.

Since CNS relapse is an uncommon event [4] it is hard to prospectively acquire tissue samples from these patients who are largely identified in a retrospective manner- CNS involvement occurs within 24 months of the diagnosis of DLBCL. Therefore, formalin-fixed paraffin-embedded (FFPE) samples are the most readily available source for molecular analysis. Two previous publications used FFPE samples in order to detect a protein expression signature that may define risk stratification for CNS relapse [18, 19]. However, FFPE preservation process leads to RNA degradation and chemical modification, which makes transcriptome analysis biologically and technically problematic. Yet, Culpin *et al.* [20] demonstrated the equality of FFPE tissues and frozen samples results with respect to miRNAs expression. Although a number of publications have demonstrated the role of miRNA in DLBCL pathogenesis [7, 21, 22] and miRNA expression was able to differentiate between subtypes of systemic DLBCLs [9, 10, 23], we could not find any published data related to the role of miRNA expression in the imminent setting of CNS relapse in newly diagnosed DLBCL. Our search for miRNAs signature that may imply CNS relapse has identified two candidates as possible mediators in this process: miR-20a and miR-30d. MiR-20a belongs to the miR-17-92 cluster, a group of six miRNAs that were found to be amplified in DLBCL [24]. MiR-17-92 cluster has been shown to suppress apoptosis and to promote cell proliferation, survival and tumorigenesis [25]. Other reports demonstrated that sustained expression of this cluster may play an important role not only in tumor formation, but also in tumor maintenance [26]. MiR-20a level was found to be increased in PCNSL compared to nodal DLBCL [27], possibly pointing on its involvement in CNS rooting. While miR-30d has no known role in DLBCL pathogenesis, it was shown to increase metastasis, apoptosis, proliferation, and differentiation [28]. In addition, miR-30d has been demonstrated to act as an oncogene in medulloblastoma [29], hepatocellular carcinoma [30] and prostate cancer [31]. It is possible that miR-30d contributes to the propensity of subsets of DLBCL to spread into the CNS since it is associated with regulation of cell proliferation and metastases.

Using prediction tools for miRNA-gene targets our analysis retrieved 241 and 35 candidate gene targets for miR-20a and miR-30d, respectably. Several bioinformatics approaches exist for interpretation of this data and turning it into biologically meaningful information. Focusing on relevant genes from the candidate list is possible by using biological pathway databases. Biological pathway databases encompass numerous well defined gene lists and allow for gene enrichment detection by answering the question “Does the experimental list represent the genes within a specific pathway more than would be expected if the set of genes were selected randomly?”. If a significantly higher number of genes originated in the experimental arm, it is reasonable to assume that the miRNA in question regulates the specific pathway. When testing for the combination of miR-20a and miR-30d overexpression using KEGG database, enrichment in several pathways is detected, including protein processing in endoplasmic reticulum (ER), Ubiquitin mediated proteolysis and Wnt signaling pathway. Mozos et al. provided preclinical evidence that interference with ER responses improves survival in DLBCL cells [32]. Pulvino et al. demonstrated that inhibition of ubiquitin-conjugating (E2) enzyme Ubc13-Uev1A inhibits the proliferation and survival of DLBCL cells [33]. Ge et al. demonstrated that Wnt/β-catenin pathway is partly activated in DLBCL and may contribute to its pathogenesis [34]. It is likely that these enriched pathways are involved in the unique properties of those clones that expand and spread to the CNS.

Another option is to use gene ontologies (i.e GO database). GO database provide controlled vocabularies of defined terms representing gene product properties as well as relations between those terms. This analysis enables looking on each gene candidate separately, focusing on its biological, molecular and cellular function. When annotating our data in GO database, several enriched pathways with “CNS signature” were detected, including axonogenesis, axon development and cell morphogenesis involved in neuronal differentiation (Table 5). Klagsbrun et al. have shown downregulation of axon development related genes in metastatic melanoma and small cell lung cancer, both with high propensity for CNS metastases [35]. Saunus et al. demonstrated enrichment of axon guidance pathway in brain metastases of breast, lung, melanoma and oesophageal cancer [36]. We assume that enrichment of pathways with CNS signature probably contribute to the propensity of DLBCL subclones for CNS homing.

Semantic similarity relates to computing the similarity between concepts, having the same meaning or related information, which are not lexicographically similar. Annotation of gene sets to MeSH data may have valuable contribution in function prediction, detection of gene-phenotype correlation and relationships between specific concepts (e.g. “gene X *is a* Wnt signaling regulator”). Using this technique, enrichment of neuronal plasticity and Jun pathway were detected (Figure 3). Current knowledge suggests that neuronal plasticity and self-renewal of stem cell like cells is partly responsible for continued tumor growth and recurrence in glioblastoma [37] and may have a similar role in DLBCL CNS relapse. Activated Jun signaling is a frequent event in DLBCL that promotes dissemination of malignant cells [38]. Downregulation of Jun dramatically reduces lymphoma cell adhesion to extracellular matrix proteins, subcutaneous tumor size in nude mice and invasive behavior. Future studies might verify involvement of these pathways in the tendency of some lymphomas to spread into the CNS. MiR-494, which was differentially expressed in discovery (NanoString) cohort, didn’t have the same trend in the validation (RT-PCR) cohort. This may be related to the biological diversity of DLBCL and emphasize the need for results validation in new samples.

The limitations of the current study include the retrospective nature of patient selection for each study cohort, the small number of tissue samples included in each clinical risk group and the lack of tissue biopsies of the relapsed disease for similar miRNA expression analysis. Our search focused on 800 microRNAs (detected via NanoString platform) out of total of over 2,000 known miRNAs. In addition, the applicability of our findings remains unknown and requires further larger scale studies.

In conclusion, miRNA expression analysis may have valuable information in stratifying DLBCL patients by their risk for CNS relapse and may shed light on the pathways involved in this complex process. Additional samples need to be obtained in order to further explore our preliminary results.

## MATERIALS AND METHODS

### Samples

Tissues were obtained from the archives of the departments of pathology at Rabin Medical Center, Meir Medical Center and Hadassah Hebrew University Medical Center. Research ethics board approval for the study had been obtained at each participating institutions. The pathology archives of the participating institutions were screened to identify newly diagnosed adult cases of DLBCL whose formalin fixed paraffin embedded (FFPE) tissue was available for evaluation. The inclusion criteria required clinical information that enabled to stratify the patients according to their risk for CNS involvement. All cases that were evaluated for the quality of the available tissue sample had either a history of secondary CNS relapse or had documented risk factors for CNS relapse. *Risk factors for CNS relapse were* based on the national comprehensive cancer network (NCCN) guidelines recommendation for CNS prophylaxis in DLBCL. We chose only cases that were diagnosed before 2013 to allow for adequate follow up periods relevant for CNS relapse. The pathological sample had to include at least a core biopsy (no fine needle aspiration or bone marrow-based diagnosis). We added available biopsies of patients with CNS relapsed lymphoma and cases of primary CNS lymphoma (PCNSL) to serve for comparison. At the final stage expert pathologists (Y.F., Y.R.) assessed 75 pathologic samples (49 systemic DLBCL, 17 primary CNS lymphoma and 9 biopsies of CNS relapsed DLBCL). The pathologist confirmed the presence of DLBCL in the FFPE samples and marked the section for areas that contain 80-90% tumor cells. Cases with low tumor cell density were excluded. Ten consecutive unstained 10 μm sections were cut from the selected biopsy blocks. Forty six FFPE tissues served for the final miRNA analysis.

### RNA extraction

FFPE samples underwent xylene/ethanol deparaffinization followed by extraction of total RNA using RecoverAll Total Nucleic Acid Isolation kit, according to manufacturer’s protocol (Ambion, Life Technology, CA, USA). Additional RNA purification was performed using phenol-chlorophorm followed by ethanol precipitation. The final RNA concentration and purity were measured using a NanoDrop ND-1000 spectrophotometer (NanoDrop Technologies, Thermo Scientific, Wilmington, USA).

### MiRNA profiling

The multiplexed NanoString nCounter miRNA expression assay (NanoString Technologies, Seattle, WA, USA) was used to profile more than 800 human miRNAs. The assay was performed according to the manufacturer’s protocol. Briefly, 100 ng of total RNA was used as input material, with 3 μL of the threefold-diluted sample. A specific DNA tag was ligated onto the 3′ end of each mature miRNA, providing an exclusive identification for each miRNA species in the sample. The tagging was performed in a multiplexed ligation reaction utilizing reverse complementary bridge oligonucleotides to dispose the ligation of each miRNA to its designated tag. All hybridization reactions were incubated at 64°C for 18 hr. Excess tags were then removed, and the resulting material was hybridized with a panel of fluorescently labeled, bar-coded reporter probes specific to the miRNA of interest. Abundances of miRNAs were quantified with the nCounter Prep Station via counting individual fluorescent barcodes and quantifying target miRNA molecules present in each sample.

### RT-PCR

Real-time polymerase chain reaction (RT-PCR) was performed to validate top significant candidates obtained by NanoString nCounter miRNA assay, according to manufacturer’s protocol (Applied Biosystems; ABI, Life Technologies, CA, USA). miRNA expression was tested using TaqMan universal PCR Master Mix (No AmpErase, UNG, ABI). PCR amplification and reading were performed using StepOne Real-Time PCR System (Life Technologies) using the following conditions: 2 min at 50°, 10 min at 95 °C followed by 40 cycles of 95 °C for 30 s and 60 °C for 1 min. Expression values were calculated based on the comparative threshold cycle method. Normalization was performed compared to miR-16 expression.

### Data analysis

All statistical analysis was conducted using R software version 3.2. NanoString data preprocessing and normalization followed by differential expression analysis was performed using DEseq2 [39] package and in house scripts. The mean value of negative controls was set as the lower threshold for each sample; only miRNAs with at least 50% of their values above the lower threshold were included in the analysis. MiRNAs displaying absolute fold-change ≥ 1.5 with a false-discovery-rate [FDR] of 5% were considered differentially expressed. RT-PCR data was analyzed using ΔΔCt analysis followed by student’s t-test. Pathway enrichment analysis was performed using clusterProfiler [12] package.

## SUPPLEMENTARY MATERIALS TABLES


