# Supplementary material for: MicroRNAs as predictors for CNS relapse of systemic diffuse large B-cell lymphoma
Source: Oncotarget. 2017 Sep 15;8(49):86020–30. doi: 10.18632/oncotarget.20902 (PMC5689664; doi:10.18632/oncotarget.20902)
Supplement: Supplementary file 1 [file oncotarget-08-86020-s001.pdf]

MicroRNAs as predictors for CNS relapse of systemic diffuse large B-cell lymphoma

SUPPLEMENTARY MATERIALS

Supplementary Table 1: miRNA-targets for miR-20a

COPS2	C20orf194	HABP2	CTNND1	UTY	SMC1A	ARHGAP17	CPEB4
RGAG4	RC3H1	KIF3B	TMEM63C	CDV3	TMEM132E	POGK	ST3GAL5
WNK3	ELFN2	MTCP1	VEZF1	XYLT1	UBE2B	USP9X	EBF1
KMT2C	MREG	CNTFR	NFAT5	UBE2W	NIPBL	ATXN1	BRAP
WSB1	RAPGEF1	PTGFRN	MMP16	ABI2	ITSN1	MTMR10	GRM1
TSPAN13	FAM114A2	SH3BP2	ACIN1	GLI3	SMAD4	CAMK2B	ANKRD34A
BBX	RAB8B	TCEANC2	PABPC1L2B	SUFU	UBN2	SYT11	PHF21A
S100A8	COL12A1	MECP2	PABPC1L2A	CALN1	ONECUT2	RLIM	INO80D
GPHN	CDCA5	SEMA6D	PELI2	RANBP17	KIAA1161	CACNA1E	SPG11
FRYL	CACNA1A	FAM168B	NFIB	AHCYL1	TMEM30B	GFRA1	SATB1
MYT1L	CCND2	CCSER2	FOXA2	GRIK3	SYNCRIP	RAB11FIP2	DAGLA
ESRRA	AEBP2	NDFIP1	PCBP4	FOXP1	NMNAT2	RP1L1	RUNX1T1
SRGAP3	POU2F1	AGO1	BCLAF1	PTCHD1	AMPH	UBFD1	RASL12
YIPF4	SH3PXD2A	CCDC85A	FAM98A	RAP2C	NUDT8	CPEB2	PTPN11
ARHGAP19	TEAD1	KIF5C	RREB1	MED13L	AMMECR1	RAB11FIP4	BTAF1
C1orf122	RCOR1	IKZF2	CRY2	CD99L2	CDC26	LAMP5	DIRAS1
CABYR	CAMK2G	CHMP7	TTLL6	GTF2H1	NRIP1	EPHA6	BTN2A2
DST	ERO1B	DROSHA	GPR83	YPEL2	MET	ATXN1L	ABHD17C
ZC2HC1C	FRAS1	TPBG	TEX19	IQCE	MIR636	LUZP6	TENM1
C1orf21	LANCL3	SLC28A3	REEP5	KLHL29	SRSF2	MTPN	PTPRU
SNHG4	HAUS5	PPP1R16B	RAB5B	TNRC6B	TOR2A	SPAST	MANEAL
PPP2CA	SHISA9	G3BP2	H2AFJ	ARID3B	MAP3K3	TANC2	KCNMA1
KDM7A	EIF5A	RPGRIP1L	TMEM260	HSD17B4	BCL2L11	MEF2A	KCMF1
CNIH2	FCHSD1	FAM167A	COG3	SLC4A4	OTUD4	CSNK1G1	AHNAK
MGEA5	ZNF317	SZRD1	ZNF521	KIAA2022	NDST1	ZNF710	TET3
GEMIN5	TSHZ1	EPHA4	GABBR2	SLC30A4	PDPK1	FRMD5	EGLN3
GAN	PLXNA4	TSPAN11	ALKBH5	MAP4	MAP2K4	ANKRD52	KCNH5
CD247	ELF3	BCKDK	RBM12B	NAA15	GTDC1	ABAT	MSL1
ZNF706	CRIM1	RFX7	MAP1B	RNF169	MAFG	TMED9	PPP1R9B
PALM2	SNPH	CCDC85C	TRIM52	GMFB	DCLK1	GPR85	MATR3
CPEB3							

Supplementary Table 2: miRNA-targets for miR-30d

FOXP1	ROBO1	ATP1B1	HEG1
APPBP2	QSER1	DNAJC6	HERPUD2
KLHL2	C11orf58	PHF12	METTL7A
CNTN4	ZNF146	RBM46	ERBIN
RAPH1	CXCL14	CNOT7	N4BP2
HOXA5	KLHL14	ARX	
RFX7	SCHIP1	TAPT1	
MYH10	HOXB13	RILPL1	
ICOS	TMEM133	USP38	
RNPS1	LRP2	KLF9	
